# Impact of Maternal Air Pollution Exposure on Children’s Lung Health: An Indian Perspective

**DOI:** 10.3390/toxics6040068

**Published:** 2018-11-16

**Authors:** Pritam Saha, Ebin Johny, Ashish Dangi, Sopan Shinde, Samuel Brake, Mathew Suji Eapen, Sukhwinder Singh Sohal, VGM Naidu, Pawan Sharma

**Affiliations:** 1 Department of Pharmacology, National Institute of Pharmaceutical Education and Research, Guwahati 781125, Assam, India; pritamsahapharm@gmail.com (P.S.); ashishdangi0@gmail.com (A.D.); sopanshinde6644@gmail.com (S.S.); vgmnaidu@niperguwahati.ac.in (V.N.); 2 Department of Pharmacy Practice, National Institute of Pharmaceutical Education and Research, Guwahati 781125, Assam, India; ebinjohny92@gmail.com; 3 Respiratory Translational Research Group, Department of Laboratory Medicine, School of Health Sciences, University of Tasmania, Launceston 7248, Tasmania, Australia; sjbrake@utas.edu.au (S.B.); mathew.eapen@utas.edu.au (M.S.E.); sukhwinder.sohal@utas.edu.au (S.S.S.); 4 Medical Sciences, School of Life Sciences, Faculty of Science, University of Technology Sydney, Sydney, NSW 2007, Australia; 5 Woolcock Emphysema Centre, Woolcock Institute of Medical Research, The University of Sydney, Sydney, NSW 2037, Australia

**Keywords:** particulate matter, air pollution, maternal-exposure, airway disease

## Abstract

Air pollution has become an emerging invisible killer in recent years and is a major cause of morbidity and mortality globally. More than 90% of the world’s children breathe toxic air every day. India is among the top ten most highly polluted countries with an average PM_10_ level of 134 μg/m^3^ per year. It is reported that 99% of India’s population encounters air pollution levels that exceed the World Health Organization Air Quality Guideline, advising a PM_2.5_ permissible level of 10 μg/m^3^. Maternal exposure to air pollution has serious health outcomes in offspring because it can affect embryonic phases of development during the gestation period. A fetus is more prone to effects from air pollution during embryonic developmental phases due to resulting oxidative stress as antioxidant mechanisms are lacking at that stage. Any injury during this vulnerable period (embryonic phase) will have a long-term impact on offspring health, both early and later in life. Epidemiological studies have revealed that maternal exposure to air pollution increases the risk of development of airway disease in the offspring due to impaired lung development in utero. In this review, we discuss cellular mechanisms involved in maternal exposure to air pollution and how it can impact airway disease development in offspring. A better understanding of these mechanisms in the context of maternal exposure to air pollution can offer a new avenue to prevent the development of airway disease in offspring.

## 1. Introduction

In recent years, air pollution has become a leading cause of morbidity, mortality, and economic loss globally [1,2]. Air pollution poses a direct and major threat to human health by inducing serious respiratory illnesses such as Chronic Obstructive Pulmonary Disease (COPD) and Asthma, and by also acting as a trigger of various forms of chronic interstitial lung diseases and lung cancer. The severity of air pollution is gauged by measuring air particulate matter (PM) [3]. PM consists of fine and coarse particulate matter; fine PM_2.5_ has an aerodynamic diameter of ≤2.5 µm, while the coarse PM_10_ is ≤10 µm. Both PM types primarily emanate from vehicles, industrial exhausts, and household sources [4]. It is well established that both PM_2.5_ and PM_10_ can cause serious respiratory damage [5]. According to a World Health Organization (WHO) report in 2018, the adverse effects produced by air pollution are responsible for 8 million premature deaths every year [6]. Air pollution contributed to at least 23% of deaths from lung cancer, 43% occurring through respiratory disease, 24% from strokes, and 23% from ischemic-heart-disease-related deaths [7]. However, India surpassed all other countries, showing the highest number of deaths due to air pollution and accounting for 2.5% of the 25% of air-pollution-related deaths globally [8]. Ninety-one percent of the world’s population inhale poor-quality air with air pollution levels that exceed the WHO guideline. Rapid industrialization and modernization further aggravate this situation by increasing airborne PM levels.

Another important factor of concern is the effect of air pollution during pregnancy, especially on fetal development. Chronic exposure to increased PM levels during pregnancy is associated with numerous adverse birth complications, such as pre-term delivery [9], low birth weight [10], birth small for the gestational age [11], congenital heart defects [12], intrauterine growth restriction (IUGR) [13,14], stillbirth [15], and spontaneous abortion [16]. Further, associated morbidities include neurodevelopmental impairment leading to attention deficit hyperactivity disorder (ADHD) and possible autism [17,18,19], neurological disorders such as impaired neurocognitive ability, nervous system sequelae [20], infant eczema [21], and increased risk of childhood asthma [22]. 

Essentially, PM is a mixture of solid and liquid particles consisting of inorganic and organic matter, dust particles, polyaromatic hydrocarbons (PAH), volatile organic compounds (VOCs), sulfates, nitrates, ammonia, and minute quantities of metals (TMs), as well as water and unidentified compounds [23]. The sources of PM can be divided into indoor and outdoor (ambient). Indoor sources include cigarette smoking, construction work, cooking with a kerosene stove or conventional wood burning (chulas), burning of incense or mosquito repellent coils, pesticides and cleaning agents used in homes, and room fresheners (aerosols) [24]. Outdoor (ambient) sources include industrial exhaust, diesel vehicles, burning of coal, wood, and road dust. Further sources of PM can be elucidated depending upon the mode of emission, such as those from anthropogenic and natural sources [25]. Anthropogenic PM sources generally would include burning of coal, petroleum products, biomass, industrial processing, agricultural activities, vehicular exhaust, and friction between tires and road, while natural sources would include volcanic eruptions, dust storms, and forest fires [26,27]. The adverse health effects of PM depend not only on the source of PM but also on the chemical composition that is adsorbed on its surface [27].

India is the ninth highest polluted country with an average PM_10_ level of 134 μg/m^3^ per year. The eight most polluted countries in descending order are as follows: Pakistan, Qatar, Afghanistan, Bangladesh, Iran, Egypt, Mongolia, and United Arab Emirates [28]. Of the 100 most polluted cities around the world, 42 are in India, the top 14 of which are listed in Table 1. Worryingly, 99% of India’s population is exposed to air pollution levels that exceed the WHO Air Quality Guideline (AQG) PM_2.5_ permissible levels of 10 μg/m^3^ [29]. Recent evidence demonstrates that on average, PM_2.5_ levels in India are found to be in the range of 10–100 μg/m^3^ [30,31]. This is highly concerning because according to the estimates by The Global Burden of Disease, PM_2.5_ exposure in India is one of the leading risk factors causing the premature death of approximately 1.67 million people [32,33]. In India, higher concentrations of PM_2.5_ are observed in northern regions rather than in the south [30,31,34]. This is because of the landlocked geographical features of the north leading to poor wind circulation, thus causing air pollutants to remain in the atmosphere for longer periods. In contrast, southern India is surrounded by coastal regions wherein sea breeze/wind plays a crucial role in pushing the pollutants away from the region. Moreover, during winters in north India, the creation of a high-pressure zone obstructs wind generation, impacting the movement of the pollutants [35]. Among metropolitan cities in India, New Delhi, Kolkata, Mumbai, and Hyderabad show the maximal PM_2.5_ levels with an average concentration of 40–81 μg/m^3^ per year, again exceeding the permissible levels (40 μg/m^3^). Air pollutants in India contribute to a decrease in life expectancy by an average of 3.4 years in the general population [36]. According to reports published by the Environmental Performance Index (EPI), approximately 3.5 billion people, i.e., half of the world’s population, are exposed to air pollutants; of these people, three-quarters live in northern region of India [37]. There have been several descriptive studies conducted in India to find the impact of air pollution in neonates and children, as represented in Table 2 [38,39,40]; however, these studies did not explore the underlying pathophysiological mechanisms. This review highlights the key prevailing issue, and as the magnitude of air pollution and its impact on human health in India was also presented and discussed at the recently held first WHO Conference on air pollution in Geneva [6], this timely review will provide stimulus to promote research efforts to investigate maternal-exposure related health effects. As scientific data demonstrate, air pollution is rapidly increasing globally, partly because of rapid urbanization, uncontrolled industrialization, and unambiguity surrounding control measures to effectively reduce it in a timely manner. It has negatively affected various aspects of human health globally (including India) which is corroborated by some recent evidence where increased burden of chronic respiratory illnesses were seen in 2016 in comparison to 1990 in India [41]. In fact, air pollution now has become the second most prominent cause of disease burden in India not only affecting lung but also affecting other major organs i.e., brain, heart, kidney. Further, India has a very young population with greater number of children. At the growing age children are very active physically (i.e., have high oxygen intake per unit body weight), they also have a faster rate of breathing and are very close to the ground where greater suspended-PM is available in the ambient air. All these factors make them vulnerable to fine-PM entering deeper into the lungs leading to both impairment in lung growth (children have greater lung surface area/kg of body mass) while their lungs are still developing [42]. In addition, PM-availability to the systemic circulation can also affect other organs negatively. The majority of the Indian population still uses biomass as a fuel to cook their food (typically 1 h of biomass-exposure is ~300 cigarette smoke, though the actual composition of both varies greatly [43,44], and biomass-exposure which is ambient air inside the house further negatively impact the health of children either directly or in utero. One of the studies found greater oxidative stress to biomass-exposure in children at 8–13 years of age and these children were more susceptible to the development of airway disease later in life [44]. Air pollution in the context of India has serious consequences later in life, such as reduced lifespan and we believe rather than focusing on the end spectrum, we must target at early stages where interventions can be most effective in reducing poor health outcomes later in life. 

This review will provide an overview of many health issues as a result of PM-exposure, both indoors and outdoors, with special emphasis on maternal exposure and its implications for children’s health in India. In this context, we also present novel cellular mechanisms underpinning the development of airway disease in children which may become future drug targets. In addition, we suggest changes in lifestyle, living/working conditions, and environmental policy prevent/reduce maternal exposure and subsequent development of airway disease in children. We found fewer clinical studies from India in this area, but collectively the cellular mechanisms of air pollution-induced poor respiratory health outcomes will largely remain same therefore some of these mechanistic insights are drawn from studies conducted globally.

## 2. Evidence of Maternal Air Pollution-Induced Health Effects in Offspring

Studies have revealed that a large number of health issues in offspring can be attributed to exposure to PM, including reduced lung function, increased chances of lower respiratory infections, cardiovascular diseases, exacerbation of chronic respiratory disease and premature mortality [37,50]. Birth weight is a key parameter to check for fetal growth. For example, it is now evident that maternal exposure to PM leads to lower birth weight (<2.5 Kg), preterm deliveries, and IUGR, which are likely factors associated with increased morbidity and mortality in offspring, as well as heightened risk of other health complications later in life [51]. PM exposure results in the generation of reactive oxygen species (ROS) and reactive nitrogen species (RNS), both of which cause oxidative stress that triggers DNA damage, leading to the development of placental DNA adduct formation [52]. In addition, one of the PM components, i.e., PAH, binds to placental growth factor receptors, resulting in an inadequate transplacental nutrient exchange. Transplacental nutrient and oxygen transport are crucial during the gestation period for regulating normal fetal growth and development [53]. Moreover, PM also contains some inorganic metals such as chromium, aluminum, silicon, titanium, iron, and copper that result in up-regulation of pro-inflammatory mediators, contributing to pulmonary inflammation [54]. Inadequate placental perfusion may also cause inflammation, resulting in growth restriction in utero due to interference with the nutrient–oxygen transfer leading to deoxygenation of both maternal blood and of the fetus [51]. It has been reported that maternal exposure to PM alters hematological parameters such as blood coagulation capacity, change in viscosity, and levels of hemoglobin, platelets, and white blood cells, which further correlates with PM toxicity leading to adverse fetal growth [55]. In addition to the changes in hematological parameters, there is also endothelial dysfunction, which is attributed to maternal PM exposure during the gestational period [56]. It has been reported that systemic inflammation and oxidative stress caused by PM exposure can increase plasma dimethylarginine concentration [57]. As dimethylarginine is an endogenous nitric oxide (NO) synthase inhibitor [58] which decreases NO levels, as a consequence, increased plasma dimethylarginine leads to endothelial dysfunction which is linked to impaired vascular function and increased risk of cardiovascular diseases. Studies also suggest that maternal exposure to PM leads to changes in a hemodynamic parameters such as an increase in blood pressure, and this may be due to stimulation of the sympathetic nerve and vasoconstriction [59]. While other studies have shown that maternal PM exposure during gestational period causes pre-eclampsia due to oxidative stress-induced changes in blood pressure homeostasis [60,61]. Prolong exposure to PM during childhood (age ranges from 3–9 years) results in reduced lung growth as well as lung function deficits in children [62,63]. Some cohort studies also demonstrate that prolong PM exposure in healthy children had a short and long-term impact on pulmonary function based on spirometry [64]. However, pollutant derived oxidative stress in pregnant mothers can block the NO-mediated vasodilatation, leading to increased blood pressure which adversely affects offspring and leads to pre-term delivery and IUGR [65]. Further, maternal PM exposure also results in inadequate fetal development [66] mainly due to abnormal blood flow to the fetus. This decrease in blood flow triggers inflammatory signals to release parturition associated cytokines, which results in membrane rupture, cervical ripening, and myometrial contraction and ultimately contributes to premature delivery [67], a plausible mechanism for PM exposure induced complications in offspring.

## 3. Maternal Air Pollution Exposure and Airway Disease

Although smoking is the greatest risk factor for the development of COPD, other exposure factors such as air pollution have emerged as significant, with the WHO estimating that 14% of premature deaths are a result of air-pollution-induced COPD globally [68]. COPD symptoms include wheezing, breathlessness, chronic cough, and further severe exacerbations, leading to reduced quality of life with a rapid decline in lung function [69,70,71,72] and risk of early mortality [73]. Asthma is one of the most prevalent pediatric chronic inflammatory respiratory diseases and is characterized by reduced airflow, airway hyperresponsiveness, and airway remodeling [74]. However, it is known that airflow obstruction in asthma is reversible while it is irreversible in COPD, mainly due to extensive loss of the small airways in COPD; this has major implications for mortality in the later stages of the disease [75]. Epidemiological studies have found a strong association between air pollution and the incidence of asthma in children [76,77]. However, many studies also indicate that these associations begin in utero [77,78,79,80]. There is strong evidence that maternal immunity plays a pivotal role in fetal–infant immune response development [81]. Maternal allergies may delay the onset of transformation to a nonallergic immune response to inhaled allergens in children, thereby increasing the chances for the development of allergic sensitization and/or asthma in the offspring [81]. Generally, lungs begin to develop during the canalicular phase, i.e., 17th–26th weeks of embryonic development. During this gestation period, the lungs are more prone to oxidative stress as antioxidant defense mechanisms are lacking. Further evidence of oxidative stress was observed in a mouse model on day 16, which resembles the canalicular stage of human pulmonary development. This model demonstrated a decrease in the number of peripheral airway branches and in alveoli formation [81].

**Figure 1:**
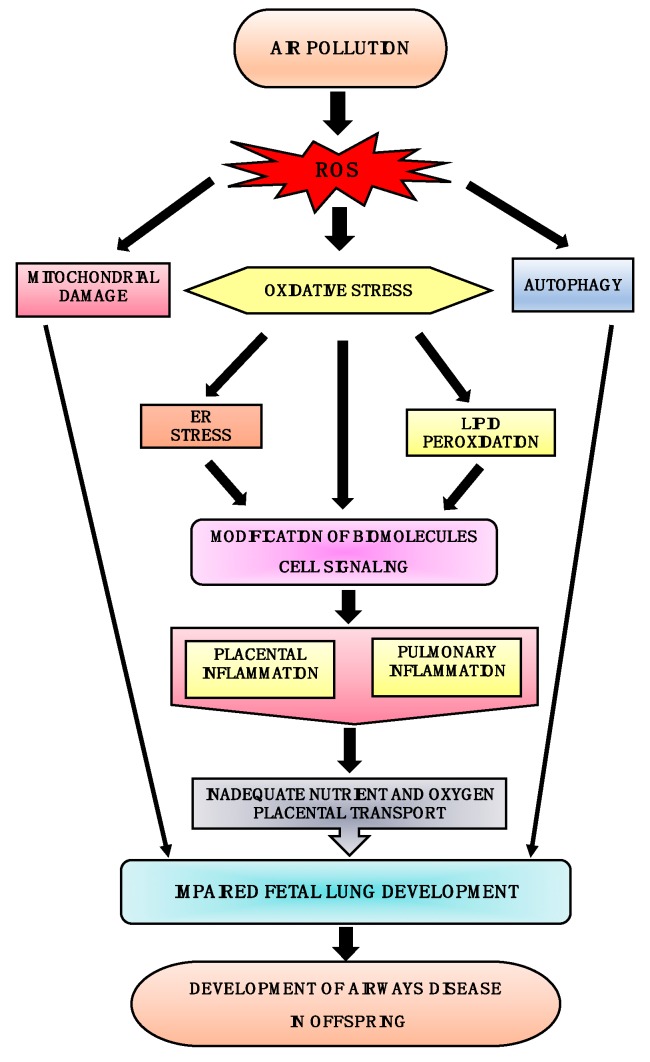
**Plausible mechanisms for the development of airway disease in offspring**. Air pollution induces ROS generation; this leads to oxidative stress followed by endoplasmic reticulum (ER) stress and lipid peroxidation, resulting in up-regulation of inflammatory genes. These cause pulmonary and placental inflammation and thereby negatively affect the nutrient and fetal oxygen transport system. Excessive ROS generation also results in mitochondrial damage and induction of autophagy. Thus, oxidative stress, ER stress, autophagy, and mitochondrial damage cause impaired fetal lung development, leading to the development of airway disease early or later in life.

It has been established that air pollution causes oxidative stress by impairing cellular endogenous antioxidant production capabilities and thus increasing ROS generation [82]. This increase in ROS leads to mitochondrial dysfunction and oxidative stress which can further damage the tissue by modifying the function of vital proteins such as NADH-ubiquinone reductase, succinate-ubiquinone reductase, cytochrome c oxidase [83]. Increases in ROS is also known to causes endoplasmic reticulum (ER) stress leading to the upregulation of inflammatory genes as well as impairment of the autophagic process, thus further aggravating the pathogenesis. Autophagy is a protective catabolic process which facilitates lysosomal-mediated degradation or clearance of misfolded or unwanted proteins. Oxidative stress causes ER stress and lipid peroxidation, resulting in mitochondrial dysfunction and pulmonary and placental inflammation; this negatively affects the nutrient and fetal oxygen transport system, ultimately leading to impairment in fetal lung development [56,84,85,86,87]. Similarly, maternal exposure to air pollution causes oxidative stress and mitochondrial impairment in the fetus which triggers pulmonary and placental inflammation and negatively affects nutrient and oxygen transport [87]. Moreover, adsorbed transition metals in air pollution further aggravate ROS generation by the process of Fenton’s reaction [83]. Fenton’s reaction leads to the subsequent activation of cellular signaling cascades and the activation of inflammatory cells and release of pro-inflammatory cytokines leading to pulmonary and placental inflammation [56,88]. Figure 1 summarizes how in uteral air pollution-induced ROS generation and subsequent cellular oxidative stress can affect a variety of cellular functions leading to impairment of fetal lung growth and ultimately the development of airway disease in children.

## 4. In Utero Cellular Mechanisms Involved in the Development of Airway Disease

**Figure 2:**
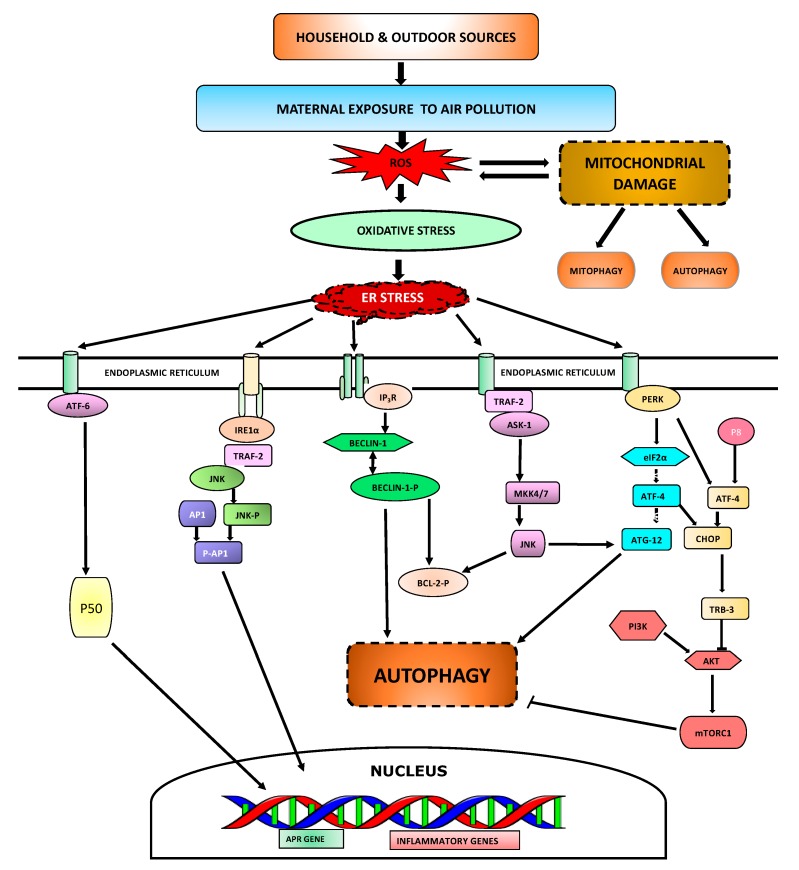
**Cellular mechanisms involved in maternal exposure to air pollution.** Air pollutants enter the lungs while breathing and increases ROS generation resulting in oxidative stress. Increase in ROS causes mitochondrial damage which can initiate cell death through various mechanisms. Oxidative stress induces ER stress that allows immunoglobulin binding protein (BiP) to bind misfolded ER proteins and release activating transcription factor-6 (ATF-6), which is then transported to Golgi apparatus and cleaved to 50 kDa form-ATF6 (p50). p50 in the nucleus drives transcription of many acute phase response (APR) and inflammatory genes. ER stress also activates inositol-requiring enzyme 1 alpha (IRE1α) kinase which recruits receptor-associated factor 2 (TRAF2) leading to the phosphorylation of Jun N-terminal kinase (JNK) and enhances activator protein-1 (AP-1)-dependent transcription of various cytokines and chemokines. On the ER membrane, inositol 1,4,5-trisphosphate receptor (IP_3_R) interacts with Beclin-1 which inhibit autophagy. However, inhibition of IP_3_R initiates detachment from Beclin-1 leading to autophagy in absence of calcium. Interaction of TRAF2 with apoptosis signal-regulating kinase 1 (ASK-1) lead to JNK activation which promotes B-cell lymphoma-2 (Bcl-2) phosphorylation, leading to detachment from Beclin-1. PERK interaction to phosphorylated EIF2α can lead to autophagy through an interaction of activating transcription factor-4 (ATF-4) dependent autophagy-related gene-12 (Atg12) protein expression. Alternatively, P8 protein interaction with ATF-4 stimulates the up-regulation of pseudokinase tribbles homolog-3 (TRB3) which further leads to activation of autophagy by inhibition of the Akt/mTORC1 complex.

Maternal exposure to air pollution affects the developing fetus and can be deleterious in terms of its impacts on the offspring’s lung health and consequential development of airway disease later in life. The cellular mechanisms/pathways involved are shown in Figure 2, and some of the key pathways are described below.

### 4.1. ER Stress

The endoplasmic reticulum (ER) is one of the key cellular organelles and plays a pivotal role in biosynthetic and signaling functions in the cell. The ER is associated with Ca^2+^ homeostasis as well as Ca^2+^-mediated signaling cascades. The ER also provides the platform for the synthesis, folding, and modification of biomolecules which are to be secreted in the plasma membrane [89,90]. These processes are assisted by various intrinsic chaperones and Ca^2+^-binding glucose-regulated proteins 78 (GRP78) or BiP (immunoglobulin heavy chain binding protein), calreticulin, calnexin, and protein folding enzymes, such as the thioredoxin-like protein disulfide isomerase (PDI) [91]. Misfolded proteins are retro-translocated to the cytosol through a process known as ER-associated protein degradation (ERAD), followed by 26S proteasomal degradation. An imbalance between ER protein folding load and capacity leads to the aggregation of misfolded proteins in the lumen, a condition that causes ER stress. The defensive or adaptive response of the ER, called Unfolded Protein Response (UPR) elements, decreases the overloaded protein synthesis to maintain ER homeostasis. The UPR elements regulate ER homeostasis in a coordinated fashion by shutting down the translation process of proteins along with a programmed gene transcriptional process to increase ER folding capacity. If this coordinated gene transcriptional process fails to provide proper ER homeostasis, then the consequential stress can induce intrinsic cellular apoptotic pathways [92]. Proteasomes are essentially responsible for clearing the accumulated misfolded protein aggregates in ER; however, when proteasome systems are dysfunctional, the UPR actively induces autophagy [92,93]. ER stress also induces the upregulation of inflammatory genes like NFkB and secretion of cytokines such as IL-23 and type I interferon [94,95]. It has been reported that GRP78 attunes UPR activation and plays a pivotal role in embryogenesis [96]. As evident from animal data, mouse pulmonary growth is initiated at Embryonic Day (E) 9.5 by the process of cell proliferation and differentiation from anterior foregut endoderm. However, the lung maturation stage is from E16.5 to postnatal Day 5, and is characterized by a decrease in cell proliferation and the formation of newly differentiated cell types, including alveolar epithelial type-1 (AT-1) and alveolar epithelial type-2 (AT-2) cells, in a distal part of the lung [97]. AT-2 cells are surfactant-producing cells which act as precursor cells for AT-1 cell synthesis (which provide the extensive surface area for gas exchange) during the lung developmental stage [98,99]. During the saccular stage of embryonic development, enhancement of surfactant protein secretion by AT-2 cells allows the lung to prepare for postnatal respiration [100]. Surfactant plays a crucial role in the reduction of surface tension; as a result, the lung can inflate with maximum capacity by increasing the compliance, thereby decreasing the work for breathing. However, AT-2 cells are regulated by ER homeostasis. An increase in ER stress results in apoptosis of AT-2 cells, thereby reducing surfactant protein secretion. Thus, ER stress can lead to respiratory dysfunction in offspring [96]. It has been reported that PERK and IREs regulate crosstalk between protective and apoptotic UPRs signaling [96]. However, modulation of PERK upon ER stress regulates cell survival signaling by reduction of the translational process via phosphorylation of Eukaryotic translation initiation factor 2a (eIF-2a) and encourages apoptosis via the PERK-eIF-2a–ATF4–CHOP pathway [101]. It has been reported that transforming growth factor (TGF)-β has a pivotal role in the regulation of apoptosis in lung epithelial cells, aside from its role in bifurcation and septae formation during pulmonary development [102]. It is also reported that ER Stress and TGF-β/Smad signaling pathways are tightly interlinked and account for apoptosis of AT-2 cells, leading to a decrease in surfactant secretion which in turn affects alveolar epithelium formation in the developing lung of offspring [96].

### 4.2. Autophagy

Autophagy means “self-eating”. It is a fundamental and highly conserved process of lysosomal degradation and recycling of misfolded proteins or damaged cytoplasmic cellular organelles to maintain cellular homeostasis [103]. The autophagic process includes engulfment of cell organelles within the vesicular membrane referred to as autophagosomes followed by lysosomal degradation with the help of lysosomal degradative enzymes [104]. It plays a crucial role in both pathological and physiological conditions such as cell survival, cellular energy production, and metabolism, as well as the innate and adaptive immune system [105,106]. Several studies have demonstrated a hereditary association of genes involved in the autophagic processes such as ULK1, SQSTM1, MAP1, LC3B, Beclin-1, and Atg5 in asthmatics. An elevated number of double-membrane structures known as “autophagosomes” were also found in fibroblast and epithelial cells in asthmatics in comparison with a healthy population [107,108]. An elevated number of autophagosomes has been reported in lung samples derived from COPD patients in comparison with healthy volunteers [109]. Under normal physiological conditions, autophagy plays a protective role in cell survival and clearance of damaged proteins, whereas in stress conditions its regulation is impaired, leading to the development of various diseases. It is now known that an altered cellular autophagic process is seen in stress conditions like asthma and COPD [103]. However, dysregulation of autophagy causes a breakdown of intracellular components to generate an energy source for extracellular matrix (ECM) production, and generation of inflammatory mediators and release of pro-fibrotic signaling molecules, resulting in airway remodeling. Furthermore, it has also been reported that selective autophagy such as mitophagy (elimination of damaged or dysfunctional mitochondria) and ciliophagy (elimination of damaged cilia or components of cilia) also comes into play in airway disease [110,111]. The transmembrane proteins Atg9 and VMP-1 [112,113] are pivotal for autophagosome formation. The assembly formed by Atg1, Atg13, and Atg17/FIP200 proteins is essential for the relocation of protein Atg9 to the autophagosome, leading to double-membrane autophagosome formation, a key indicator for autophagy determination. However, the mammalian target of rapamycin complex1 (mTORC1) regulates autophagy by repressing the activity of assembly proteins Atg1–Atg13–Atg17/FIP200; thus, inhibition of mTORC1 induces the initiation of the autophagic process. Furthermore, rapamycin, which blocks mTORC1, results in the induction of autophagy. Bafilomycin A1 molecule inhibits fusion with autophagosomes and inhibits vacuolar-type H^+^-ATPase, leading to the impairment of lysosomal degradation capability [114]. The kinase Akt, which is upstream of regulators of mTORC1, phosphorylates and inactivates TSC2 and thereby Akt activation stimulates mTORC1 and inhibits autophagy. Increased autophagy in the context of airway disease acts as a pro-survival factor required for the normal recycling process of damaged proteins or organelles, and there is an increase in autophagic markers in sputum and peripheral blood cells in asthma [115].

Autophagy plays a pivotal role in embryogenesis as the autophagic process is initiated during the fertilization phase from two-cell to eight-cell division. A defect in Beclin-1 protein leads to embryonic death (E10–E14) with the impairment of neural tube formation [116]. As evident from clinical data, during the early gestation period, i.e., 7–11 weeks, interstitial extra-villous trophoblast (EVT) cells occupy the placental side, and a trophoblastic plug restricts the maternal blood flow, resulting in a hypoxic condition. Moreover, during 12–16 weeks, endovascular EVT dilates the blood vessels in spiral arteries, leading to an elevation in intervillous space blood flow [117]. Under normal conditions, there is an increase in autophagic flux to meet physiologic demand whereas under pathological conditions, decreased autophagy flux results in impairment of lysosomal-dependent degradation. However, autophagic inhibition in trophoblasts induces inadequate placental blood supply in the initial phase of the gestation period, leading to a hypoxic condition in the embryo. Furthermore, release of anti-angiogenic factors from villi due to hypoxic conditions aggravates dysregulation of trophoblasts by inhibiting the autophagic process.

Thus, autophagic inhibition causes impairment of homeostasis in trophoblast cells. Controlled regulation of the autophagic process is necessary for reproduction. However the dysregulation of functional autophagy impacts offspring health because inadequate placental blood flow affects lung development in offspring [56,117]. In this way, dysregulation of autophagy leads to impaired lung development, ultimately leading to the development of airway disease.

### 4.3. Mitochondrial Damage

Mitochondria are the leading subcellular organelle from which ROS are generated under physiological conditions during catalyzation of ATP production, which is linked to the electron transport chain that occurs in the inner mitochondrial membrane. The mitochondrial respiratory chain has four complexes in the inner membrane where electrons move in a synchronous manner, i.e., move from a high to a low redox potential, resulting in the generation of mitochondrial membrane potential [118,119,120]. Air pollution which has both organic and inorganic compounds is capable of generating ROS which interfere in the electron transfer chain system by disrupting the Q cycle that operates between complexes I and III, resulting in mitochondrial superoxide production [121,122]. Air pollutants also have the ability to disrupt mitochondrial permeability transition pore [123], a crucial molecule that regulates mitochondrial respiration and regulates cellular apoptosis in a controlled manner [124,125]. However, disruption of the mitochondrial outer membrane by air pollutants leads to the release of several pro-apoptotic proteins such as cytochrome c, second mitochondria-derived activator of caspases (SMAC), and apoptosis-inducing factor (AIF) into the cytosol where they activate apoptotic signaling pathways, ultimately leading to programmed cell death [125,126]. Besides the direct effects of air pollutants on mitochondria, the physiology of this organelle can also be altered indirectly by increasing ROS generation and Ca^2+^ flux in the cell. Even free intracellular calcium [Ca^2+^]_i_ plays an important role in regulating the opening and closing of the mitochondrial permeability transition pore [127]. ROS such as H_2_O_2_ can induce a rise of [Ca^2+^]_i_ in various cells by either inhibiting sarco/endoplasmic reticulum Ca^2+^-ATPase (SERCA) or plasma membrane Ca^2+^-ATPase (PMCA), leading to the activation of 1, 3, 5-trisphosphate (IP_3_) receptors [128,129]. Various constituents in air pollution can directly target the mitochondrial membrane and cause structural damage [83]. The process in which nonfunctional or damaged mitochondria are cleared via mitochondria-selective autophagy is known as ‘”mitophagy” and has been reported as one of the key mechanisms in the development of airway disease [130].

## 5. Conclusion

Air pollution has become a growing concern in recent years in India and globally, causing an enormous burden to society as well as loss of life. Air pollutants, particularly PM_2.5_, penetrate deep into the lungs and cause pathological changes which lead to impaired pulmonary function. Epidemiological studies demonstrate that exposure to PM induces oxidative stress that leads to serious health effects. Similar mechanisms are involved in maternal air-pollution-exposure-induced health effects on offspring that are a result of increased oxidative stress resulting in damage to proteins and biomolecules and causing placental inflammation, induction of ER stress–autophagy, and mitochondrial dysfunction. Therapeutic strategies targeting air-pollution-induced oxidative stress and related signaling should be considered to enhance endogenous antioxidant defense mechanisms in the cell. On the other hand, use of novel autophagy modulators that can also block ER stress could be of great value for future research into preventing the development of pollutant-induced airway disease. Collectively, air-pollution-induced adverse health outcomes can be prevented by adopting a comprehensive global approach that works locally to eliminate or reduce the burden of PM both indoors and outdoors.

## Figures and Tables

**Table 1:** List of the top 14 most polluted Indian cities [45].

RANK	CITY	PM_2.5_ LEVEL (Annual Mean, µg/m^3^)
1	Kanpur	173
2	Faridabad	172
3	Varanasi	151
4	Gaya	149
5	Patna	144
6	Delhi	143
7	Lucknow	138
8	Agra	131
9	Muzaffarpur	120
10	Srinagar	113
11	Gurgaon	113
12	Jaipur	105
13	Patiala	101
14	Jodhpur	98

**PM:** Particulate matter (Diameter of ≤2.5 µm).

**Table 2:** List of studies on air pollution that have been carried out in an Indian population.

S. No.	Author	Study Design	Sample Size	Exposure	Parameter Studied	Comments and Association
1.	Padhy et al., 2009 [44]	Case-control	Control (105) Biomass user (115)	Biomass smoke	Respiratory symptoms Oxidative stress Hematological changes	Exposure to biomass smoke significantly associated with respiratory diseases, oxidative stress, and hematological changes
2.	Awasthi et al., 2010 [42]	Cohort	23 children (10–13 years of age)	Agriculture crop residue burning (ACRB)	Pulmonary function	Decrease in pulmonary function with an increase in air pollutant levels due to ACRB
3.	Kumar et al., 2015 [46]	Cohort	3104 children	Indoor suspended particulate matter (SPM)	Asthma	Indoor SPM level was significantly higher in asthmatic children’s houses
4.	Singh et al., 2015 [47]	Cross-sectional, multicenter	44,928 (6–7 year age group); 48,088 (13–14 year age group)	Traffic pollution, maternal and paternal smoking	Asthma	Traffic pollution and maternal and paternal smoking is associated with increased prevalence of asthma
5.	Murlidhar et al., 2015 [48]	Case-report	11-year-old boy, malnourished	Secondary exposure to sandstone mining	Silico-tuberculosis	Mother started working in the mines soon after her marriage and the family lives close to the mines
6.	Rumchev et al., 2017 [49]	Cohort	170 children between 1 and 15 years	Indoor exposure to PM_2.5_	Respiratory symptoms	No significant association between PM-exposure and respiratory symptoms even though odds are high
